# Do fungal pathogens exploit HLA-G-mediated immune tolerance?

**DOI:** 10.3389/fmolb.2026.1963030

**Published:** 2026-10-16

**Authors:** Abdullah AlSaleh, Eman Farid

**Affiliations:** Department of Microbiology, Immunology and Infectious Diseases, College of Medicine and Health Sciences, Arabian Gulf University, Manama, Bahrain

**Keywords:** fungal infections, HLA-G, IL-10, immunosuppression, mycosis, TGF

## Abstract

Fungal pathogens employ diverse strategies to evade host immunity and establish persistent infection, yet the contribution of immune checkpoint pathways to fungal pathogenesis remains incompletely understood. Human leukocyte antigen-G (HLA-G) is a nonclassical major histocompatibility complex class I molecule with potent immunoregulatory properties. It is capable of suppressing innate and adaptive immune responses through interactions with inhibitory receptors. Although HLA-G-mediated immune tolerance is increasingly recognized in cancer, transplantation, and persistent viral and parasitic infections, its role in fungal disease remains largely unexplored. This review examines the hypothesis that fungal pathogens may exploit HLA-G-mediated immune tolerance to facilitate infection and persistence. Particular attention is given to interleukin-10 and transforming growth factor-β, two immunoregulatory cytokines linked to HLA-G expression that are prominently induced during various fungal infections. The convergence of these pathways provides a mechanistic framework connecting fungal-induced immune regulation with potential HLA-G activity. Establishing this relationship could reveal an underrecognized mechanism of fungal immune evasion and identify opportunities for host-directed therapy.

## Introduction

Fungal diseases encompass a pathological spectrum ranging from superficial and mucosal infections to life-threatening invasive mycoses, with clinical outcomes determined by the interplay between fungal virulence and the host immune response. Protective immunity against fungal pathogens is orchestrated by a network of innate and adaptive immune cells, including neutrophils, monocytes, macrophages, dendritic cells, NK cells, and antigen-specific T lymphocytes, which cooperate to recognize fungal cell-wall ligands, prevent tissue dissemination, and promote durable immune protection (Lionakis et al., 2023). Nevertheless, pathogenic fungi have evolved multiple strategies to circumvent these defenses, such as concealing pathogen-associated molecular patterns (PAMPs), switching morphotypes, resisting phagocytic killing, reshaping myeloid cell responses, and inducing regulatory cytokine networks that attenuate antifungal inflammation (Lionakis et al., 2023).

Among the diverse immunoregulatory mechanisms that maintain immune homeostasis, increasing evidence indicates that pathogens can exploit host immune checkpoint pathways to attenuate protective immunity (Khanna et al., 2016; DiPalma et al., 2025). One such pathway involves the nonclassical major histocompatibility complex (MHC) class I molecule human leukocyte antigen-G (HLA-G) (DiPalma et al., 2025; Rizzo et al., 2014). It is a tolerogenic nonclassical HLA class I molecule that is recognized as a modulator of immune homeostasis in pregnancy, transplantation, autoimmunity, cancer, and infection. This immune-modulation role gained keen interest in the context of viral infections, as it exhibits dual functionality. On the one hand, HLA-G is associated with dampening cytotoxic T-cell responses, weakening antigen-presenting cell function and attenuating natural killer-cell cytotoxicity (Beli et al., 2025). On the other hand, it serves as a protective feedback mechanism that restrains tissue injury during excessive antiviral inflammation (Beli et al., 2025). It is worth noting that conventional HLA-G expression is restricted to immune-privileged tissues where foreign antigens are tolerated without triggering a destructive inflammatory response; however, under pathophysiological conditions, elevated neo-expression of HLA-G is often detected (Gonzalez et al., 2012).

The HLA-G locus resides within the major histocompatibility complex on chromosome 6p21.3. Alternative splicing generates at least seven canonical isoforms, four membrane-bound isoforms (HLA-G1 to HLA-G4) and three soluble isoforms (HLA-G5 to HLA-G7) (Carosella et al., 2008; Ishitani and Geraghty, 1992). HLA-G1 and HLA-G5 are the best characterized because they preserve a classical class I-like architecture and typically associate with β2-microglobulin, whereas shorter isoforms lack one or more extracellular domains with variations in affinity potential and biochemical behavior (Carosella et al., 2008; Lin and Yan, 2018). The principal receptors identified for HLA-G are leukocyte immunoglobulin-like receptor B1 (LILRB1/ILT2/CD85j) and LILRB2 (ILT4/CD85d), which are expressed on an extended spectrum of lymphoid and myeloid populations (Carosella et al., 2008; Kuroki et al., 2019; Shiroishi et al., 2006). Additionally, KIR2DL4 has also been implicated as an HLA-G-associated receptor in selected settings, especially in NK cell biology (Rajagopalan and Long, 2012). These receptors often exhibit heterogeneous affinity toward different HLA-G isoforms; for example, LILRB1 preferentially recognizes β2-microglobulin-associated HLA-G, whereas LILRB2 can bind more permissively to β2-microglobulin-free forms (Kuroki et al., 2019; Shiroishi et al., 2006). This heterogeneous affinity and contingent heterogeneous expression patterns may play a significant role in the resulting phenotype of the immune response (Krijgsman et al., 2020).

HLA-G-mediated immune regulation in fungal infections may have context-dependent consequences shaped by the interplay between pathogen-associated characteristics and host immune responses, which is consistent with the principles of the damage-response framework (DRF) of microbial pathogenesis (Casadevall and Pirofski, 2003; Pruksaphon et al., 2024). In that context, increased HLA-G activity in an immunocompromised host could further suppress protective antifungal responses, thereby promoting fungal persistence and pathogen-associated damage. Conversely, in the setting of an excessive inflammatory response, HLA-G induction could represent a compensatory mechanism that limits immune-mediated tissue injury. The balance between these outcomes is likely to vary with host immune status, fungal species, anatomical site, and stage of infection.

Unlike conventional fungal immune-evasion strategies that predominantly interfere with discrete antimicrobial mechanisms, HLA-G functions as a broad-spectrum immune checkpoint capable of simultaneously suppressing the activity of NK cells, antigen-presenting cells, and both CD4^+^ and CD8^+^ T lymphocytes (Beli et al., 2025). Given its well-established immunoregulatory role in infection, transplantation, and cancer, HLA-G represents a compelling candidate pathway through which fungal pathogens may promote immune tolerance and persistence. In this review, we examine the molecular mechanisms by which fungal pathogens may induce HLA-G expression and discuss how HLA-G-mediated immune regulation could facilitate fungal survival.

## Biomarkers upregulating HLA-G

Although there is limited evidence demonstrating HLA-G induction during fungal infections, numerous studies have identified molecular pathways implicated in regulating HLA-G expression, many of which are prominent features of host responses to fungal pathogens. In that sense, it is postulated that fungal infections may create a microenvironment that is highly permissive for HLA-G expression, providing a mechanistic basis by which fungi could exploit host immune tolerance and facilitate persistence. The proposed pathway, its current evidentiary gaps, and its alternative immunological consequences are illustrated in Figure 1.

**FIGURE 1:**
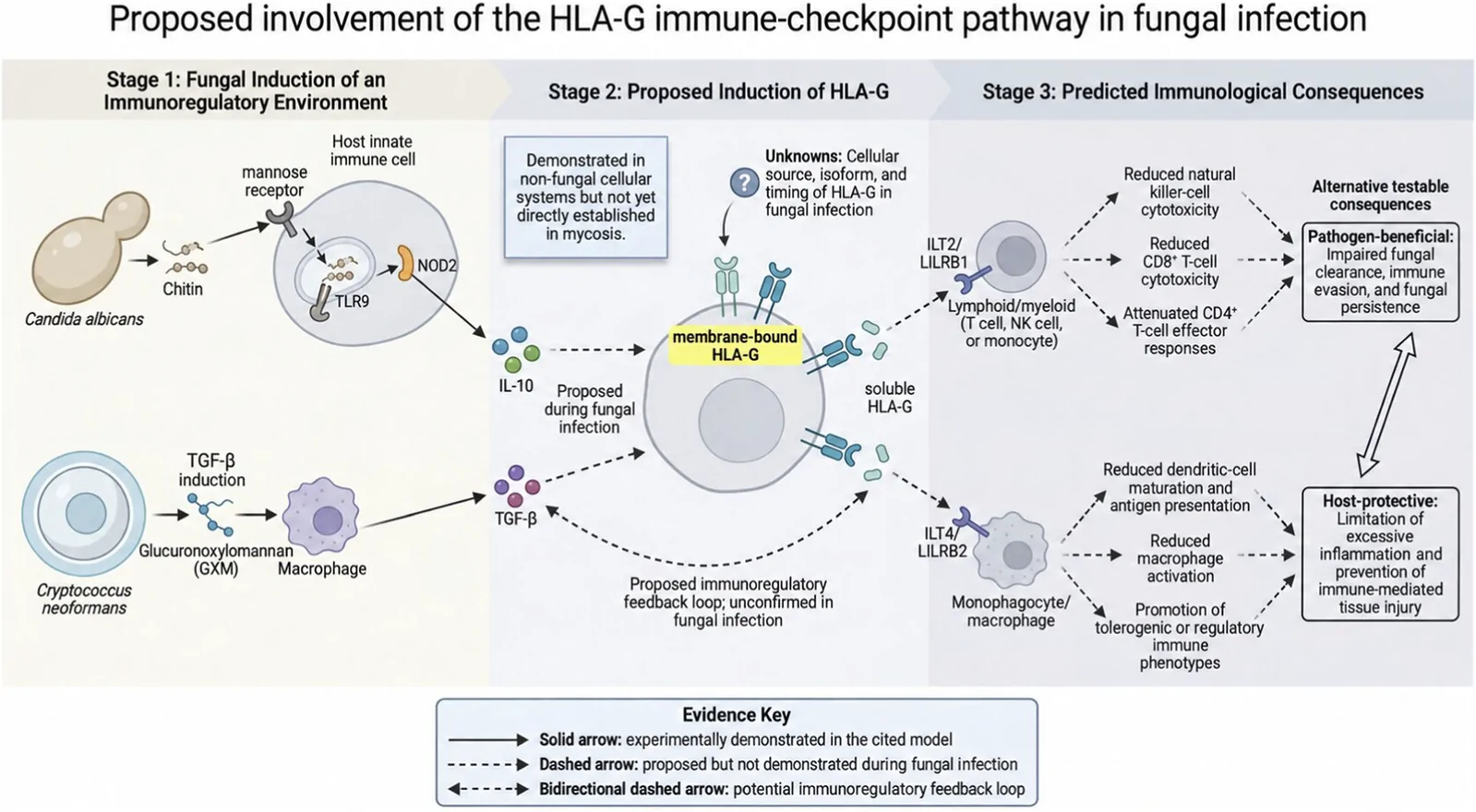
Proposed involvement of HLA-G-mediated immune tolerance in fungal infection. Fungal organisms and defined fungal components can induce host immunoregulatory responses involving IL-10 and TGF-β. These cytokines have independently been shown to upregulate HLA-G in nonfungal cellular systems, providing a potential but unconfirmed mechanistic link between fungal infection and HLA-G expression. Membrane-bound and soluble HLA-G could engage the inhibitory receptors ILT2/LILRB1 and ILT4/LILRB2 on lymphoid and myeloid cells, thereby attenuating antifungal effector functions. Such regulation could facilitate fungal immune evasion and persistence or, alternatively, represent a compensatory host response that limits inflammation-mediated tissue injury. Dashed arrows indicate relationships that remain to be demonstrated in fungal infection.

Interleukin-10 (IL-10) represents the strongest mechanistic link between fungal infection and HLA-G induction. As a pleiotropic anti-inflammatory cytokine produced by monocytes, macrophages, dendritic cells, regulatory T cells, and other immune cells, IL-10 plays a central role in limiting excessive inflammation while promoting immune tolerance (Moore et al., 2001). Direct experimental evidence has demonstrated that IL-10 selectively upregulates HLA-G transcription and protein expression in human trophoblasts and peripheral blood monocytes (Moreau et al., 1999). Moreover, IL-10 promotes the differentiation of tolerogenic DC-10 cells expressing both HLA-G and ILT4, while HLA-G can further enhance IL-10 production, establishing a self-amplifying immunoregulatory feedback loop that suppresses antigen presentation and effector lymphocyte function (Amiot et al., 2014a). This IL-10-HLA-G axis is consistently observed across infectious diseases; for instance, increased circulating IL-10 correlates with elevated soluble HLA-G levels in chronic hepatitis C and human cytomegalovirus (hCMV) infection, and similar cytokine-driven HLA-G upregulation has been described in HIV, hepatitis B, and several parasitic infections (Amiot et al., 2014a; Weng et al., 2011; Amiot et al., 2014b; Yan et al., 2009; Lajoie et al., 2009; Donaghy et al., 2007). Collectively, these findings support the notion that IL-10 is a conserved upstream regulator of HLA-G across multiple infectious diseases.

Immunity against fungal pathogens elicits both pro-inflammatory and immunoregulatory responses; indeed, balancing these responses ultimately determines whether the host achieves fungal clearance, limits inflamed tissue injury, or develops persistent infection. Among the produced regulatory cytokines, IL-10 plays a central role in constraining excessive inflammation while simultaneously suppressing antigen presentation, macrophage activation, and Th1/Th17 effector responses that are critical for antifungal immunity (Romani, 2011). In *Candida albicans* infection, IL-10 is produced by monocytes, macrophages, dendritic cells, and regulatory T cells following fungal recognition, particularly during chronic mucosal colonization and sustained antigenic stimulation (Netea et al., 2015). One of the best-characterized mechanisms involves fungal chitin, an inner cell wall polysaccharide that becomes increasingly exposed during cell wall remodeling, budding, and antifungal-induced cell wall damage (Netea et al., 2015). Purified *C. albicans* chitin induces IL-10 production through coordinated signaling involving the mannose receptor, nucleotide-binding oligomerization domain-containing protein 2 (NOD2), and Toll-like receptor 9 (TLR9), thereby suppressing inflammatory responses in both *in vitro* and *in vivo* models (Netea et al., 2015; Wagener et al., 2014). In contrast, neutralization of IL-10 during experimental systemic candidiasis enhanced macrophage nitric oxide production and significantly improved resistance in susceptible mice, demonstrating that excessive IL-10 compromises protective antifungal immunity (Romani et al., 1994; Netea et al., 2004).

Moreover, a comparable immunoregulatory profile has been described in cryptococcosis (Scriven et al., 2016). In patients with HIV-associated cryptococcal meningitis, elevated circulating IL-10 was independently associated with increased cerebrospinal fluid fungal burden and innate immunity deactivation (Scriven et al., 2016). These immune abnormalities correlated with circulating levels of cryptococcal glucuronoxylomannan (GXM), and exposure of human monocytes to purified GXM reproduced features of this deactivated phenotype *in vitro*, suggesting that cryptococcal capsular components actively promote an IL-10-dominated immunoregulatory state rather than merely reflecting advanced disease (Scriven et al., 2016). Similarly, elevated IL-10 levels have been associated with increased susceptibility to invasive aspergillosis, particularly in immunocompromised hosts, including hematopoietic stem cell transplant recipients and patients receiving prolonged corticosteroid therapy, where excessive immunoregulation contributes to impaired fungal clearance (Romani, 2011). Interestingly, in a murine model of post sepsis aspergillosis, blockade of the PD-1 immune checkpoint enhanced fungal clearance with a correlation to reduced IL-10 secretion by antigen-presenting cells and T lymphocytes (Vu et al., 2020).

Given that IL-10 is one of the most potent physiological inducers of HLA-G, the IL-10-rich microenvironment generated during mycosis provides a compelling mechanistic basis for HLA-G induction as an additional layer of immune regulation; however, more investigations are needed to establish a direct association in the context of fungal infections.

Transforming growth factor-β (TGF-β) represents another immunoregulatory mediator supporting the potential involvement of HLA-G in fungal infection. TGF-β is a pleiotropic cytokine that dampens inflammatory responses by promoting regulatory T-cell differentiation and modulating the effector functions of both innate and adaptive immune cells (Mao et al., 2023; Rashidi et al., 2022; Mestrallet et al., 2021). Furthermore, TGF-β and HLA-G participate in a reciprocal immunoregulatory relationship, whereby TGF-β can enhance HLA-G expression, while HLA-G can in turn promote TGF-β production (Brugiere et al., 2015; Guan et al., 2015; Gregori et al., 2010). Thus, sustained TGF-β responses during infection may provide a favorable immunological environment for the emergence and reinforcement of HLA-G-mediated immune tolerance (Mestrallet et al., 2021). The relevance of this relationship to infection is supported by viral and parasitic diseases, in which manipulation of TGF-β signaling is a well-recognized component of pathogen-host interactions (Rashidi et al., 2022; Mirzaei and Faghihloo, 2018; Drewry and Harty, 2020). Notably, both viral and parasitic infections are also associated with altered HLA-G expression, supporting the broader concept that TGF-β-rich infectious environments may coexist with and potentially reinforce HLA-G-mediated immune tolerance (Rashidi et al., 2022).

Moreover, TGF-β involvement has been documented across several fungal infections. In *Candida* infections, for instance, evidence shows activation of TGF-β/Smad signaling in *C. albicans*-associated endometrial fibrosis in Asherman’s syndrome, increased TGF-β production during experimental vaginal candidiasis, and mobilization of platelet-derived TGF-β during *Candida*-induced airway inflammation (Taylor et al., 2000; Zhao et al., 2023; Wang et al., 2025). In cryptococcosis, the major capsular polysaccharide glucuronoxylomannan (GXM) has been shown to induce TGF-β secretion by macrophages, while in a cryptococcosis rat model, TGF-β reduced antibody- and serum-mediated phagocytosis of *Cryptococcus neoformans* by alveolar macrophages and peripheral blood monocytes, as well as reduced chemokine production and impaired fungal clearance (Villena et al., 2008; Shao et al., 2005). Additionally, elevated circulating TGF-β levels have been reported in patients with chromoblastomycosis, with levels declining during clinical improvement but subsequently rising in association with persistent fungal lesions and progressive fibrosis (da Silva et al., 2010). Collectively, these observations associate TGF-β as a recurrent component of the immunoregulatory microenvironment of fungal infections. Given its capacity to enhance HLA-G expression and its established interplay with HLA-G in other persistent infections, fungal-induced TGF-β provides an additional mechanistic basis for HLA-G-mediated immune tolerance. However, a direct link between fungal-induced TGF-β and HLA-G expression remains to be demonstrated.

Representative empirical studies supporting the individual links between fungal infection, IL-10/TGF-β, and HLA-G are summarized in Table 1.

**TABLE 1:** Representative empirical evidence supporting individual components of the proposed fungal–IL-10/TGF-β–HLA-G pathway.

Pathway examined	Experimental settings	Related findings	Evidentiary interpretation	References
IL-10 → HLA-G	Cultured human trophoblasts and peripheral blood monocytes treated with IL-10	IL-10 increased HLA-G transcription in trophoblasts and increased HLA-G transcription and cell-surface expression in monocytes	Direct evidence that IL-10 can induce HLA-G in these human cells	(Moreau et al., 1999)
Fungal chitin → IL-10	Human PBMCs, murine bone-marrow-derived macrophages, receptor-deficient cells, and mouse inflammation models exposed to purified fungal chitin	Chitin derived principally from *C. albicans* induced IL-10 through pathways involving the mannose receptor, NOD2, and TLR9 and attenuated LPS-induced inflammation in mice	Direct evidence that a purified fungal component can induce IL-10	(Wagener et al., 2014)
IL-10 → susceptibility to candidiasis	Susceptible DBA/2 mice challenged with attenuated *C. albicans* PCA-2 and treated with anti-IL-10 antibodies	IL-10 neutralization prevented mortality and was associated with increased IFN-γ-stimulated macrophage NO production *ex vivo* and enhanced Th_1_ responses	Functional evidence that IL-10 contributed to susceptibility in this experimental model	(Romani et al., 1994)
GXM/cryptococcosis ↔ IL-10-rich immune phenotype	Prospective cohort of patients with HIV-associated cryptococcal meningitis	Higher serum GXM titers correlated with serum IL-10 and a mortality-associated immune signature that included monocyte deactivation and impaired Th1 responses	Clinical evidence of association between cryptococcal antigen burden, IL-10, immune dysfunction, and mortality	(Scriven et al., 2016)
TGF-β → HLA-G	BGC823 and SGC7901 human gastric cancer cells treated with recombinant TGF-β	TGF-β increased HLA-G mRNA and soluble HLA-G through suppression of miR-152; miR-152 restoration attenuated this effect	Direct mechanistic evidence that TGF-β can induce HLA-G in gastric cancer cells	(Guan et al., 2015)
Cryptococcal GXM → TGF-β	RAW 264.7 and murine peritoneal macrophages exposed to purified cryptococcal capsular polysaccharides	GXM predominantly induced TGF-β secretion; GXM and GalXM separately induced Fas/FasL-mediated macrophage apoptosis	Direct evidence that a purified cryptococcal component can induce TGF-β	(Villena et al., 2008)
TGF-β → altered cryptococcal immunity	Rat macrophage and monocyte experiments and experimental pulmonary cryptococcosis	TGF-β inhibited phagocytosis, chemokine production, and oxidative burst but enhanced lysozyme-mediated fungal control; early treatment increased fungal burden, whereas treatment during chronic infection reduced it	Direct evidence that TGF-β has timing- and context-dependent effects during cryptococcosis	(Shao et al., 2005)

## Conclusion

Collectively, the evidence reviewed here proposes a plausible role for HLA-G in fungal immune evasion, although direct demonstration remains limited. The convergence of HLA-G-mediated immune suppression with fungal induction of immunoregulatory pathways, particularly IL-10 and TGF-β, provides a compelling framework in which HLA-G may contribute to the establishment and persistence of fungal infection. Future studies should therefore determine whether HLA-G is induced during mycosis, define its cellular source and molecular form, and establish whether fungal-induced IL-10 and TGF-β directly regulate its expression, as in viral and parasitic infections. Resolving these questions would clarify whether HLA-G represents a previously underrecognized component of fungal pathophysiology rather than merely an associated marker of immune dysregulation.

Confirmation of this pathway could also reveal new therapeutic opportunities. HLA-G and its inhibitory receptors ILT2 and ILT4 are already being investigated as immune checkpoint targets in oncology, raising the possibility that selective blockade could similarly restore antifungal immune function. Given the delicate balance between protective immunity and inflammation-mediated tissue damage during mycoses, any intervention should favor transient, local or receptor-selective modulation rather than indiscriminate immune activation and should complement, rather than replace, effective antifungal therapy. Ultimately, establishing whether the HLA-G axis is functionally exploited by fungal pathogens may identify a previously unrecognized immune checkpoint and a potential target for adjunctive host-directed antifungal therapy.
